# Erratum to ‘Causal association between subtypes of osteoarthritis and common comorbidities: A Mendelian randomisation study’ [Osteoarthritis and Cartilage Open 5 (2023) 100414]

**DOI:** 10.1016/j.ocarto.2025.100660

**Published:** 2025-10-26

**Authors:** Will Thompson, Subhashisa Swain, Sizheng Steven Zhao, Anne Kamps, Carol Coupland, Changfu Kuo, Sita Bierma-Zeinstra, Jos Runhaar, Michael Doherty, Weiya Zhang

**Affiliations:** a Academic Rheumatology, Clinical Sciences Building, Nottingham City Hospital, Hucknall Road, Nottingham, NG5 1PB, United Kingdom; b Nuffield Department of Primary Care Health Sciences, Radcliffe Primary Care Building, Radcliffe Observatory Quarter, Woodstock Road, Oxford, OX2 6GG, United Kingdom; c Centre for Epidemiology Versus Arthritis, Division of Musculoskeletal and Dermatological Sciences, The University of Manchester, Manchester, United Kingdom; d Department of General Practice, Erasmus MC University Medical Center Rotterdam, Dr. Molewaterplein 40, 3015 GD Rotterdam, the Netherlands; e Centre for Academic Primary Care, School of Medicine, University Park, Nottingham, NG7 2RD, United Kingdom; f Division of Rheumatology, Allergy and Immunology, Chang Gung Memorial Hospital, 5, Fu-Hsing Street, Taoyuan, 333, Taiwan, ROC

The publisher regrets that the file for the “supplementary material” actually contained a replica of the main text. We have now changed that file to the true supplementary material. We would also like to mention that the original submission missed an important acknowledgement to UK Biobank. The following acknowledgement has now been added; “This research has been conducted using the UK Biobank Resource under Application Number 75427. This work uses data provided by patients and collected by the NHS as part of their care and support. Copyright© 2023, NHS England. Re-used with the permission of the NHS England and UK Biobank. All rights reserved.”.

The publisher would like to apologise for any inconvenience caused.

