# Supplementary material for: Causal association between subtypes of osteoarthritis and common comorbidities: A Mendelian randomisation study
Source: Osteoarthr Cartil Open. 2023 Oct 21;5(4):100414. doi: 10.1016/j.ocarto.2023.100414 (PMC10630649; doi:10.1016/j.ocarto.2023.100414)
Supplement: Multimedia component 1 [file mmc1.docx]

Introduction

Osteoarthritis(OA) is the most common cause of arthritis worldwide and affects 10% of adults in the UK [1]. It is the second most common cause of musculoskeletal pain after back pain[2], and is a major health burden within the UK [3].

OA is a commonly diagnosed comorbidity with other chronic health conditions such as cardiovascular, musculoskeletal, and neuropsychiatric conditions in the UK and other European countries [4]. In our previous research, we performed latent class analysis with electronic health data from the Clinical Practice Research Database (CPRD) to identify categories (or clusters) of comorbidities among patients with existing OA. We were able to classify people with OA into four broad clusters (cardiovascular, musculoskeletal and mental health, musculoskeletal and cardiovascular, and metabolic) based on their comorbidities, as well as a “relatively healthy” cluster [5]. One of the emerging clusters found was musculoskeletal and/or chronic pain with mental illness. The cluster includes conditions such as fibromyalgia, depression, chronic fatigue, irritable bowel syndrome (IBS) and gout. We have also previously investigated the association of chronic conditions with incident OA recording in primary care using data from the CPRD (using age, sex and practice matched controls without OA), finding that individuals with pre-existing comorbidities were more likely to develop OA within the following 20 years (an association being found for 40 of the 49 comorbidities assessed) [6]. It was discovered that fibromyalgia and polymyalgia were the leading comorbidities associated with knee OA within 20 years, and gout and irritable bowel syndrome were the leading comorbidities associated with ankle/foot OA within 20 years. The association between OA and fibromyalgia and the aforementioned conditions (with the exception of gout) is consistent with a central pain sensitisation hypothesis of OA (where OA diagnosis is driven by pain perception) as opposed to a peripheral mechanism hypothesis (where OA is due to accumulated damage to the joints) [7].

The reason for the frequent co-occurrence of OA with comorbidities remains largely unknown. It may be because of shared risk factors such as ageing, or because of OA itself, the comorbidities themselves or the medication used. However, using observational studies to investigate such concurrence always runs the risk of bias due to unmeasured confounding. An alternative method for estimating causal effects is Mendelian Randomization (MR), where genetic variants are used as instrumental variables for a given exposure, which are randomly assigned and are not influenced by later environmental confounders. However, this rests on the assumption that the genetic variants used *are* valid instrumental variables for the exposure being measured [8]. OA is represented differently at different joints, for example OA in the hand is different from knee OA. The association of different joint specific OA with comorbidities is not yet well studied.

Even though we previously explored the bidirectional association between OA and different comorbidities using an epidemiological observational study design, the causal relationship is difficult to explain [6]. We therefore decided to perform a bidirectional MR analysis to investigate the causal relationship between three subtypes of symptomatic OA (Knee, Hip and Hand OA) and the five least studied comorbidities, namely fibromyalgia (which we proxied with multisite chronic pain, a core symptom of fibromyalgia [9]), depression, fatigue (which we proxied with self-reported tiredness [10]), IBS and gout (which we included in order to proxy peripheral mechanisms of OA).

Materials and Methods

This study used a Two-Sample MR framework, where genetic variants proxying the exposure are identified in one population sample, and their effects on an outcome are tested in a separate sample, as well as making use of available summary statistics from GWAS. MR hinges on three core assumptions, specifically: (i) the genetic variants are robustly associated with the exposure being measured; (ii) the genetic variants are not associated with known confounding variables; and (iii) the genetic variants only affect the outcome through the exposure (i.e., no horizontal pleiotropy) [8]. This study is part of an international collaborative project on Comorbidities in Osteoarthritis (ComOA) [11].

*Main analysis*

*Data Sources*

*OA*

This analysis used the most recent and largest GWAS of OA to identify the genetic instruments used, which to date is the Genetics of OA (GO) Consortium meta-analysis [12]. GO Consortium includes UK Biobank as a contributing cohort. The consortium data includes participants from European and East Asian (1% of the Knee OA sample, independent of UK Biobank) ancestry populations from the UK, Iceland, Estonia, Japan, the Netherlands, Greece, China, the USA and Norway from 1984 onwards. Participants were selected from a range of cross-sectional and longitudinal studies and registries, and OA cases were defined as symptomatic (i.e., self-reported joint pain), radiographic and hospital diagnosed (i.e., ICD-10 codes) OA. In total, GWAS from 13 cohorts contributed to the meta-analysis, with the summary single nucleotide polymorphism (SNP) values being pooled using a fixed effects inverse variance weighting (IVW) method. All studies were approved by local research ethics committees. The GO Consortium performed GWAS for multiple OA phenotypes, and this analysis used the following as exposures: Knee OA (62,497 cases, 333,557 controls), Hip OA (N= 35,445 cases, 316,943 controls) and Hand OA (20,901 cases, 282,881 controls; defined as OA in the carpometacarpal joints) [12]. We chose not to use results from the All OA (N = 177,517 cases, 649,173) GWAS, which included patients with any OA phenotype as cases [12], as OA pathology is mechanistically different per joint site [13], potentially leading to causal effects cancelling each other out.

*Comorbidities*

We reported findings of five comorbidities, namely fibromyalgia (which we proxied with multisite chronic pain, a core symptom of fibromyalgia [9]), depression, fatigue (which we proxied with self-reported tiredness [10]), IBS and gout in this manuscript. The ComOA consortium is also exploring associations with other conditions, not of interest to this manuscript.

To test the effect of comorbidities on OA, we selected genetic instruments from the largest available GWAS that had published its results in a peer reviewed journal, or was made available for external analysis, up until July 2022.

A summary of the different data sources used for this study can be found in Table 1.For depression, the largest published GWAS did not report the summary statistics, so the identified SNPs could not be used for MR directly [14]. Therefore, to select genetic instruments, we used the Howard et al 2019 GWAS (246,363 cases, 561,190 controls) [15] which was a meta-analysis of three earlier GWAS, including UK Biobank [15]. To proxy fatigue, we used frequency of tiredness [10]. The largest published GWAS of frequency of tiredness only reported one genome-wide significant SNP [10], hence we instead used an unpublished GWAS of frequency of tiredness from the Medical Research Council-Integrative Epidemiology Unit (MRC-IEU) GWAS pipeline [16]. To proxy fibromyalgia, we used multisite chronic pain [9]. The largest available GWAS for multisite chronic pain was Johnson et al 2019 [17]. For IBS the largest available GWAS was the Eijsbouts et al 2021 GWAS (53,400 cases, 433,201 controls) [18], which was a meta-analysis of three GWAS from two cohorts, including UK Biobank. The UK Biobank cohort was split between IBS questionnaire responders and non-responders to reduce confounding [18]. For gout, we used the unpublished 2018 GWAS of gout based on self-reported non-cancer illness codes from the MRC-IEU GWAS pipeline [16].

*Statistical Analysis Methods*

*Instrumental variable selection*

We extracted the genetic instruments from the genome-wide data set for the exposure being measured. Of the genome wide significant (p < 5e^-8^) SNPs extracted from the data set, we used LD clumping to identify the lead SNPs, excluding all non-independent SNPs at a threshold of r^2^ < 0.001 within an LD window of 1000 kb. For datasets accessible on the MRC IEU database, this was achieved in R (version 4.1.2) using the TwoSampleMR R package [16]. For publicly available datasets that were not available on the MRC IEU database, this was achieved using the “ieu-gwas-r” package [19].

To ensure that our effect estimates represented the effect of increased exposure risk on an outcome, all SNP associations were harmonized to the exposure increasing allele before analysis[20]. SNPs that are both palindromic and indistinguishable allele frequencies (MAF >0.499) were excluded from the analyses, as were SNPs that were not available in all data sets. When MR was performed, SNPs were weighted by their reported exposure association, Steiger filtering was applied to remove SNPs that explained greater variance for outcome than exposure [21]. To perform Steiger filtering, we estimated the F-statistic for each SNP association.

After performing the analysis, we estimated the minimal detectable odds ratio for an effect on the outcome, given 80% power, p-value of 0.05, the exposure GWAS sample size and the total variance explained by the genetic instruments [22].

*Inverse Variance Weighted (IVW) method*

Once the instrumental variables were selected, we independently extracted individual SNP-outcome associations and individual SNP-exposure associations from the data sets. These associations were measured either in the natural-log odds ratio (logOR), or per unit change. We then calculated the ratio of the SNP-outcome association over the SNP-exposure association (i.e., Wald ratios) and pooled the results by joint site (Knee OA, Hip OA and Hand OA). In addition, we also pooled the Wald ratio results by each comorbidity (i.e., all OA SNPs effect on the comorbidity, and the comorbidity SNPs effect on all joint sites simultaneously) to get the Average Causal Effect of/on OA. We used the TwoSampleMR package [16] to estimate both the individual and the overall causal effects using random effects IVW analysis (the random effects model being used to control for between SNP heterogeneity). For the individual Wald ratios, we presented them on forest plots to examine the heterogeneity visually and to present I^2^ values that we estimated from the IVW analyses Q statistics. Where the exposure was in logOR, the overall causal estimate was multiplied by 0.693 (i.e., log (2)), to be equivalent to the change per 2-fold increase in the binary exposure [23]. Where the exposure was per unit change, the original measure was used, hence equivalent to the change per 1 unit change increase in the exposure.

*Sensitivity Analysis*

In Two Sample MR, there is a risk that if the two samples have an overlapping population, the MR result will be biased towards the confounded association when using weak instruments. This is an issue when the GO Consortium GWAS includes UK Biobank, and the selected comorbidity GWAS datasets are 45-100% UK Biobank participants. However, if the genetic instruments are strong (F-statistic >10) then this bias is less likely to be an issue [24], therefore we used the GO Consortium meta-analysis *with* UK Biobank (which produced more genome-wide significant hits) as the main result. Nonetheless, as a sensitivity analysis, we independently used GWAS data sets from the GO Consortium meta-analysis GWAS *without* UK Biobank (Knee OA, 42,996 cases, 255,278 controls; Hip OA, 25,159 cases, 271,589 controls; Hand OA, 19,385 cases, 276,817 controls) [12] for both the OA to comorbidities and comorbidities to OA MR analyses. Note, as this was a sensitivity analysis for the main IVW estimates, we chose not to present the MR-Egger and weighted median estimates for this analysis.

In addition, we undertook two other methods to verify the results, specifically: i) MR-Egger, which uses the SNP-exposure and SNP-outcome associations as equivalent to points for linear regression, with the intercept value being used to determine the presence of horizontal pleiotropy [25]; and ii) weighted median analysis, where the median value for all the estimates is selected to reduce the impact of weak instruments (providing less than 50% of the variants are weak instruments) [26].

*Patient and public involvement*

Three PPI representatives with OA were involved in this study through group meetings. They provided their inputs at each step of the study, including discussions about the struggles of living with multiple conditions, the lack of research in causal relationship and management of multimorbidity, and the importance for this study. The results of the study were shared with the members in lay-person language and their inputs were considered in writing the manuscript.

Results

We performed bi-directional MR, estimating the causal effects both of OA on comorbidities (Figure 1) and comorbidities on OA (Figure 2). Of the participants included in our analysis, > 99% came from European ancestry backgrounds in developed countries. At maximum there was a 25% overlap between the exposure and outcome (main Knee OA with tiredness, multisite chronic pain and gout). Details of the minimum odds ratios and mean F statistics for each analysis can be found in Supplementary Table 1. All the analysis performed had a mean F statistic greater than 10 (range = 33.49 to 133.78; Supplementary Table 1).

*Effect of OA on comorbidities*

There was inconsistent evidence of a causal effect of Knee OA on depression, multisite chronic pain, and gout (Figure 3). There was no causal effect of any subtype of OA on tiredness or IBS. However, there was consistent evidence (i.e., p-value < 0.05 for both the main and sensitivity analyses) of a causal effect of Hip OA on multisite chronic pain (Figure 4). No evidence of causal effects was seen for Hand OA (Figure 5), and there was a consistent Average Causal Effect of OA on multisite chronic pain and inconsistent evidence for a causal effect on tiredness (Figure 6).

Supplementary Table 2 presents the SNP-associations for this analysis, Supplementary Figures 1-5 present additional forest plots of the causal effects and Supplementary Figures 6-15 present the Wald ratio effects for the SNPs used.

*Effect of comorbidities on OA*

There was consistent evidence of a causal effect of depression on Knee OA, Hand OA, and Pooled OA, but inconsistent evidence of a causal effect on Hip OA (Figures 3-6). There was consistent evidence of a causal effect of tiredness on Knee OA, Hand OA, and an Average Causal Effect on OA (Figures 3, 5 and 6). There was consistent evidence of a causal effect of multisite chronic pain on all OA subtypes and an Average Causal Effect on OA (Figures 3-7). There was no causal effect of IBS on any subtype of OA. There was inconsistent evidence of a protective effect of gout on Hip OA and an Average Causal Effect on OA (Figure 4 and 6).

Supplementary Table 3 presents the SNP-associations for this analysis, Supplementary Figures 16-18 present additional forest plots of the causal effects and Supplementary Figures 19-27 the Wald ratio effects for the SNPs.

*Other sensitivity analyses*

For the MR-Egger analysis, there was no evidence of horizontal pleiotropy for most of the consistent causal effects in the main analysis, apart from the effect of multisite chronic pain on Hand OA, where pleiotropy was detected, and the direction of effect changed. There was also evidence of a protective effect of Hand OA on depression and multisite chronic pain when adjusting for pleiotropy, and a protective effect of IBS on all OA subtypes.

For the weighted median analysis, all the consistent causal effects from the main analysis were replicated, apart from the effect of depression and tiredness on Hand OA (though the point estimates were still consistent with the main causal effects). Evidence was also found for an effect of Knee OA on multisite chronic pain, IBS and gout, Hand OA on tiredness and gout on Hip OA.

Supplementary Table 4 presents the results for fixed effects IVW, MR-Egger and the weighted median analysis.

Discussion

We used two sample MR methods to investigate bidirectional causal relationships between subtypes of symptomatic OA and five common comorbidities. The key findings are: (i) there was a bidirectional causal association between Hip OA and multisite chronic pain, (ii) there was consistent evidence of causal effect of multisite chronic pain on all types of OA, and (iii) there was a causal effect of depression and tiredness on Knee OA and Hand OA. The findings were supported by the sensitivity analysis with and without the UK Biobank data, as well as supported by the other two MR analyses, apart from the horizontal pleiotropy bias observed for Hand OA. Some consistent evidence was also observed for an Average Causal Effect on OA for depression, tiredness and multisite chronic pain, but that needs to be taken with caution due to the heterogeneity between, and double counting SNPs for the different phenotypes of OA.

The general finding of the effects of comorbidities on OA being stronger than the effects of OA on comorbidities is consistent with a previous study by Barowsky et al 2021 [27] for depression. The evidence of a causal effect of Hip OA on multisite chronic pain is consistent with cross-sectional research which found that 58% of patients diagnosed with radiographic Hip or Knee OA (rather than purely symptomatic OA) reported low back pain and pain at peripheral sites [28]. Further evidence of a causal effect of Hip OA on chronic pain comes from the observation that hip replacement is associated with long term change in brain structures linked to pain processing [29]. The relatively weak evidence of a causal relationship in our study between Hip OA and tiredness contrasts with earlier observations of an association between fatigue and Hip OA [30]. The finding of multisite chronic pain having a stronger effect on OA than of OA on multisite chronic pain is counter to studies which suggest that OA (particularly Knee OA) occurs before diagnosis of fibromyalgia [6, 31]. Similarly, previous studies have observed that prior diagnosis of Knee OA is associated with later diagnosis of depression [32], which was contradicted by our findings. However, these are all observational studies, either cross-sectional or cohort design. The former can only define association whereas the latter can only define temporality, and none can define causality.

Large estimates detected for depression, tiredness and multisite chronic pain on OA is consistent with an important role of central pain sensitisation in symptomatic OA and the association with depression and anxiety and other pain traits [33]. Pain sensitisation has been shown to have a genetic component [17, 34], meaning it varies between individuals. It is hypothesized that people with more pain sensitisation are more likely to be diagnosed earlier than those with less pain sensitisation [7]. A central pain sensitivity mechanism could potentially be explained with the delta-sleep deprivation hypothesis, leading to pain sensitisation and an increased diagnosis of fibromyalgia and OA [35]. However, other studies have suggested that general sleep quality is more strongly associated with fibromyalgia and OA than any specific pattern of sleep [36]. Another possible mediator of the effects of depression, tiredness and multisite chronic pain on OA could be BMI. BMI has been shown to be strongly associated with depression [17, 37], fatigue [38] and multisite chronic pain [17, 39], as well as having a direct causal effect on Knee OA due to increased joint strain [40].

For the effect of Hip OA on multisite chronic pain, it is possible that pain and biomechanical changes associated with Hip OA cause secondary biomechanical insults to other lower limb joints and to the lumbar spine. This may lead to additional pain at these sites and has been suggested as a likely explanation for the association between Hip OA and lower back pain [41].

*Strengths and Limitations*

This study used the largest available GWAS of OA, including the largest non-UK Biobank GWAS of OA for the sensitivity analysis [12]. All our genetic instruments had an average F-statistic greater than 10, limiting the risk of weak instrument bias [24]. However, there were numerous estimates that were inconsistent between the full meta-analysis GWAS data set and the data set without UK Biobank. Given that the full GWAS meta-analysis includes UK Biobank (2% for Hand OA, 16% for Hip OA and 25% for Knee OA), there may be bias linked to overlapping samples and weak instruments for these estimates. The only causal effect of OA on a comorbidity was Hip OA on multisite chronic pain. However, more SNPs could be identified for Hip OA than for the other joint sites, meaning a greater amount of variance in Hip OA could be explained, hence the lack of associations could be due to statistical power. There was also some evidence of horizontal pleiotropy in our estimates, namely estimates for Hand OA and irritable bowel syndromes effects on outcomes. The uncertainty in the effect of comorbidities on Hand OA is consistent with the finger, thumb and wrist joints being non-load bearing, making any pain in these regions less noticeable [42], consistent with the central pain hypothesis. For IBS, it should be noted that the IBS variant with the largest negative association with OA (rs12549729; CLDN23) was not reported in the original GWAS due to quality control issues [18]. Whilst there is the possibility that much of the effects detected in this study could be mediated by BMI, it is beyond the scope of this study, which is focussed on specific comorbidities, to investigate. Depression, tiredness and multisite chronic pain are traits that are strongly correlated with each other, thus there is a potential risk of violation of the second assumption of MR. Whilst we did not attempt to identify the effects of these comorbidities independently from each other, this could be achieved using multivariate MR methods [43]. Also, as a large percentage of the GO consortium GWAS of OA [12] is based on self-reported symptomatic OA, this study is less relevant to understanding the causal relationship of comorbidities with OA pathology. This study was conducted using data derived from predominately (> 99%) European ancestry populations, thus the findings (particularly those for depression and OA) may not replicate in African [44] or Asian [45] ancestry populations.

Given the evidence found in this study for a causal effect of comorbidities linked to central pain sensitisation on all types of OA, it is possible that psychological interventions to help patients deal with pain could be effective at reducing the pain due to OA [46]. However, due to the strong correlation between the central pain comorbidities and BMI [17, 37-39], further research is needed to differentiate the effects of central mechanisms (i.e., multisite chronic pain, depression, and fatigue) from the genetically correlated peripheral mechanisms (i.e., BMI).

In conclusion, we have found consistent evidence of bidirectional causal effect of Hip OA and multisite chronic pain, consistent evidence of a causal effect of multisite chronic pain on all types of OA, and depression and tiredness on Knee and Hand OA. However, this is just the first step to investigate the causality between OA and five common comorbidities. Further study is needed to confirm the results and to understand the mediating pathways of these causal relationships and potential targets for further treatment.

Acknowledgements

We are grateful to all the staff at the University of Nottingham who have assisted in this project, including the IT service team who facilitated our use of the High-Performance Computing (HPC) system for this study. We also wish to thank the Foundation for Research in Rheumatology (FOREUM) who funded and supported the project, and the FOREUM Patient Research Partners, Stevie Vanhegan, Jenny Cockshull and Irene Pitsllidou, who contributed to discussions concerning the relevance, quality and validity of our research. We would like to thank the GO Consortium, for providing us with the GWAS datasets for OA, in particular the datasets for the OA GWAS without UK Biobank.

Author Contributions

WZ, MD and WDT designed this study, with further development coming from CFK, SS and SZ. WDT, WZ, SS and AK wrote the statistical analysis plan, based on a previous analysis plan written by AK. WDT undertook most of the analyses with support from WZ, MD, CC, AK, SZ and SS. SBZ and JR supervised AK in her work. WDT wrote the first draft of the paper with support from WZ, MD, CC, SZ and SS; all authors read and made critical revisions to the paper. WDT, WZ, MD, CC, SZ and SS are guarantors of the papers integrity.

Ethics Statement: This study used publicly available summary data and did not involve contact with participants; thus, no extra ethical approval or informed consent was required. The research complies with the Declaration of Helsinki.

Data Availability Statement: This study uses two-sample MR, using summary statistics from predominately publicly available GWAS datasets.

The summary statistics for the main GO Consortium GWAS of osteoarthritis were downloaded from the Musculoskeletal Knowledge Portal (mskkp.org) in March 2022 from <https://msk.hugeamp.org/dinspector.html?dataset=Boer2021_OA_Mixed_Main>

The summary statistics for the sensitivity GO Consortium GWAS without UK Biobank were requested directly from the GO Consortium steering committee via email to [go-sc@listen.helmholtz-muenchen.de](mailto:go-sc@listen.helmholtz-muenchen.de) and the data was downloaded in April 2022

The summary statistics for depression, tiredness and gout were downloaded using the R package TwoSampleMR from the IEU Open GWAS project between March and August 2022; <https://gwas.mrcieu.ac.uk/>

The summary statistics for multisite chronic pain from the Johnston et al 2019 were downloaded via the University of Glasgow Enlighten website from doi:10.5525/gla.researchdata.822

The summary statistics for irritable bowel syndrome from Eijsbouts et al 2021 were downloaded via the NHGRI-EBI GWAS catalog from <http://ftp.ebi.ac.uk/pub/databases/gwas/summary_statistics/GCST90016001-GCST90017000/GCST90016564/>

Funding Disclosure: As part of this study, CC, AK and WZ applied for and received funding from the Foundation of Research for Rheumatology (2019-2022). WZ has also received funding from Versus Arthritis (grant no. 21595 and 20777), the Football Association regarding Foot and ankle Osteoarthritis and Cognitive impairment in retired UK Soccer players (FOCUS), the Medical Research Council regarding the Alleviate Pain Data Hub (MR/W014335/1), and the National Institute for Health Research for the Allopurinol Treat to Target clinical trial and the Research for Patient Benefit fund (19023). SSZ is supported by a National Institute for Health Research Clinical Lectureship and works in centres supported by Versus Arthritis (grant no. 21173, 21754 and 21755).

Conflict of Interests Statement: MD has received consultation fees from AstraZeneca, Grunenthal, and Mallinckrodt. WZ has received consultation fees from AstraZeneca, Grunenthal, Eli Lilly and Regeneron advisory boards, and speaker fees from Xiangya Hospital and Shenzhen Rheumatological Meeting. The other authors declare no competing interests.

References

1. Swain S, Sarmanova A, Mallen C, Kuo CF, Coupland C, Doherty M, et al. Trends in incidence and prevalence of osteoarthritis in the United Kingdom: findings from the Clinical Practice Research Datalink (CPRD). Osteoarthr Cartil. 2020;28(6):792-801. doi: <https://doi.org/10.1016/j.joca.2020.03.004>.

2. Macfarlane GJ, Jones GT, Hannaford PC. Managing low back pain presenting to primary care: where do we go from here? Pain. 2006;122(3):219-22. Epub 2006/04/25. doi: 10.1016/j.pain.2006.03.013. PubMed PMID: 16630692.

3. WORKING WITH ARTHRITIS: Arthritis Research UK; 2016 [cited 2022 10th of May]. Available from: <https://www.versusarthritis.org/media/2071/working-with-arthritis-policy-report.pdf>.

4. Kamps A, Runhaar J, de Ridder MAJ, de Wilde M, van der Lei J, Zhang W, et al. Occurrence of comorbidity following osteoarthritis diagnosis: a cohort study in the Netherlands. Osteoarthr Cartil. 2023;31(4):519-28. doi: 10.1016/j.joca.2022.12.003.

5. Swain S, Coupland C, Strauss V, Mallen C, Kuo CF, Sarmanova A, et al. Clustering of comorbidities and associated outcomes in people with osteoarthritis - A UK Clinical Practice Research Datalink study. Osteoarthr Cartil. 2022;30(5):702-13. doi: <https://doi.org/10.1016/j.joca.2021.12.013>.

6. Swain S, Coupland C, Mallen C, Kuo CF, Sarmanova A, Bierma-Zeinstra SMA, et al. Temporal relationship between osteoarthritis and comorbidities: a combined case control and cohort study in the UK primary care setting. Rheumatology. 2021;60(9):4327-39. doi: 10.1093/rheumatology/keab067.

7. Clauw DJ, Hassett AL. The role of centralised pain in osteoarthritis. Clin Exp Rheumatol. 2017;35 Suppl 107(5):79-84. Epub 2017/10/03. PubMed PMID: 28967359.

8. Lawlor DA. Commentary: Two-sample Mendelian randomization: opportunities and challenges. Int J Epidemiol. 2016;45(3):908-15. doi: 10.1093/ije/dyw127.

9. Sarzi-Puttini P, Giorgi V, Marotto D, Atzeni F. Fibromyalgia: an update on clinical characteristics, aetiopathogenesis and treatment. Nat Rev Rheumatol. 2020;16(11):645-60. doi: 10.1038/s41584-020-00506-w.

10. Deary V, Hagenaars SP, Harris SE, Hill WD, Davies G, Liewald DCM, et al. Genetic contributions to self-reported tiredness. Mol Psychiatry. 2018;23(3):609-20. doi: 10.1038/mp.2017.5.

11. Swain S, Kamps A, Runhaar J, Dell, Isola A, Turkiewicz A, et al. Comorbidities in osteoarthritis (ComOA): a combined cross-sectional, case–control and cohort study using large electronic health records in four European countries. BMJ Open. 2022;12(4):e052816. doi: 10.1136/bmjopen-2021-052816.

12. Boer CG, Hatzikotoulas K, Southam L, Stefánsdóttir L, Zhang Y, Coutinho de Almeida R, et al. Deciphering osteoarthritis genetics across 826,690 individuals from 9 populations. Cell. 2021;184(18):4784-818.e17. Epub 2021/08/28. doi: 10.1016/j.cell.2021.07.038. PubMed PMID: 34450027; PubMed Central PMCID: PMCPMC8459317.

13. Deveza LA, Loeser RF. Is osteoarthritis one disease or a collection of many? Rheumatology (Oxford). 2018;57(suppl_4):iv34-iv42. Epub 2017/12/22. doi: 10.1093/rheumatology/kex417. PubMed PMID: 29267932; PubMed Central PMCID: PMCPMC6251697.

14. Levey DF, Stein MB, Wendt FR, Pathak GA, Zhou H, Aslan M, et al. Bi-ancestral depression GWAS in the Million Veteran Program and meta-analysis in >1.2 million individuals highlight new therapeutic directions. Nat Neurosci. 2021;24(7):954-63. doi: 10.1038/s41593-021-00860-2.

15. Howard DM, Adams MJ, Clarke T-K, Hafferty JD, Gibson J, Shirali M, et al. Genome-wide meta-analysis of depression identifies 102 independent variants and highlights the importance of the prefrontal brain regions. Nat Neurosci. 2019;22(3):343-52. doi: 10.1038/s41593-018-0326-7.

16. Hemani G, Zheng J, Elsworth B, Wade KH, Haberland V, Baird D, et al. The MR-Base platform supports systematic causal inference across the human phenome. eLife. 2018;7:e34408. doi: 10.7554/eLife.34408.

17. Johnston KJA, Adams MJ, Nicholl BI, Ward J, Strawbridge RJ, Ferguson A, et al. Genome-wide association study of multisite chronic pain in UK Biobank. PLOS Genet. 2019;15(6):e1008164. doi: 10.1371/journal.pgen.1008164.

18. Eijsbouts C, Zheng T, Kennedy NA, Bonfiglio F, Anderson CA, Moutsianas L, et al. Genome-wide analysis of 53,400 people with irritable bowel syndrome highlights shared genetic pathways with mood and anxiety disorders. Nat Genet. 2021;53(11):1543-52. doi: 10.1038/s41588-021-00950-8.

19. Elsworth B, Lyon M, Alexander T, Liu Y, Matthews P, Hallett J, et al. The MRC IEU OpenGWAS data infrastructure. bioRxiv. 2020:2020.08.10.244293. doi: 10.1101/2020.08.10.244293.

20. Hartwig FP, Davies NM, Hemani G, Davey Smith G. Two-sample Mendelian randomization: avoiding the downsides of a powerful, widely applicable but potentially fallible technique. Int J Epidemiol. 2016;45(6):1717-26. Epub 2017/03/25. doi: 10.1093/ije/dyx028. PubMed PMID: 28338968; PubMed Central PMCID: PMCPMC5722032.

21. Hemani G, Tilling K, Davey Smith G. Orienting the causal relationship between imprecisely measured traits using GWAS summary data. PLOS Genet. 2017;13(11):e1007081. doi: 10.1371/journal.pgen.1007081.

22. Burgess S. Sample size and power calculations in Mendelian randomization with a single instrumental variable and a binary outcome. Int J Epidemiol. 2014;43(3):922-9. Epub 2014/03/13. doi: 10.1093/ije/dyu005. PubMed PMID: 24608958; PubMed Central PMCID: PMCPMC4052137.

23. Burgess S, Labrecque JA. Mendelian randomization with a binary exposure variable: interpretation and presentation of causal estimates. Eur J Epidemiol. 2018;33(10):947-52. Epub 2018/07/25. doi: 10.1007/s10654-018-0424-6. PubMed PMID: 30039250; PubMed Central PMCID: PMCPMC6153517.

24. Burgess S, Davies NM, Thompson SG. Bias due to participant overlap in two-sample Mendelian randomization. Genet Epidemiol. 2016;40(7):597-608. Epub 2016/10/19. doi: 10.1002/gepi.21998. PubMed PMID: 27625185; PubMed Central PMCID: PMCPMC5082560.

25. Bowden J, Davey Smith G, Burgess S. Mendelian randomization with invalid instruments: effect estimation and bias detection through Egger regression. Int J Epidemiol. 2015;44(2):512-25. Epub 2015/06/08. doi: 10.1093/ije/dyv080. PubMed PMID: 26050253; PubMed Central PMCID: PMCPMC4469799.

26. Bowden J, Davey Smith G, Haycock PC, Burgess S. Consistent Estimation in Mendelian Randomization with Some Invalid Instruments Using a Weighted Median Estimator. Genet Epidemiol. 2016;40(4):304-14. Epub 2016/04/12. doi: 10.1002/gepi.21965. PubMed PMID: 27061298; PubMed Central PMCID: PMCPMC4849733.

27. Barowsky S, Jung J-Y, Nesbit N, Silberstein M, Fava M, Loggia ML, et al. Cross-Disorder Genomics Data Analysis Elucidates a Shared Genetic Basis Between Major Depression and Osteoarthritis Pain. Front Genet. 2021;12. doi: 10.3389/fgene.2021.687687.

28. Hoogeboom TJ, den Broeder AA, Swierstra BA, de Bie RA, van den Ende CHM. Joint-pain comorbidity, health status, and medication use in hip and knee osteoarthritis: A cross-sectional study. Arthritis Care Res (Hoboken). 2012;64(1):54-8. doi: <https://doi.org/10.1002/acr.20647>.

29. Rodriguez-Raecke R, Niemeier A, Ihle K, Ruether W, May A. Brain gray matter decrease in chronic pain is the consequence and not the cause of pain. J Neurosci. 2009;29(44):13746-50. Epub 2009/11/06. doi: 10.1523/jneurosci.3687-09.2009. PubMed PMID: 19889986; PubMed Central PMCID: PMCPMC6666725.

30. Fu K, Makovey J, Metcalf B, Bennell KL, Zhang Y, Asher R, et al. Sleep Quality and Fatigue are Associated with Hip Osteoarthritis Pain Exacerbations: An Internet-Based Case-Crossover Study. J Rheumatol. 2019:jrheum.181406. doi: 10.3899/jrheum.181406.

31. Mahgoub MY, Elnady BM, Abdelkader HS, Abdelhalem RA, Hassan WA. Comorbidity of Fibromyalgia in Primary Knee Osteoarthritis: Potential Impact on Functional Status and Quality of Life. Open Access Rheumatol. 2020;12:55-63. Epub 2020/05/23. doi: 10.2147/oarrr.S252748. PubMed PMID: 32440236; PubMed Central PMCID: PMCPMC7212986.

32. Zheng S, Tu L, Cicuttini F, Zhu Z, Han W, Antony B, et al. Depression in patients with knee osteoarthritis: risk factors and associations with joint symptoms. BMC Musculoskelet Disord. 2021;22(1):40. doi: 10.1186/s12891-020-03875-1.

33. Akin-Akinyosoye K, Frowd N, Marshall L, Stocks J, Fernandes GS, Valdes A, et al. Traits associated with central pain augmentation in the Knee Pain In the Community (KPIC) cohort. Pain. 2018;159(6):1035-44. Epub 2018/02/14. doi: 10.1097/j.pain.0000000000001183. PubMed PMID: 29438225; PubMed Central PMCID: PMCPMC5959005.

34. Burri A, Ogata S, Rice D, Williams FMK. Twelve-year follow-up of chronic pain in twins: Changes in environmental and genetic influence over time. Eur J Pain. 2018;22(8):1439-47. doi: <https://doi.org/10.1002/ejp.1233>.

35. Doherty M, Smith J. Elusive 'alpha-delta' sleep in fibromyalgia and osteoarthritis. Ann Rheum Dis. 1993;52(3):245. Epub 1993/03/01. doi: 10.1136/ard.52.3.245-b. PubMed PMID: 8484686; PubMed Central PMCID: PMCPMC1005032.

36. Yeung WK, Morgan K, McKenna F. Comparison of sleep structure and psychometric profiles in patients with fibromyalgia, osteoarthritis and healthy controls. J Sleep Res. 2018;27(2):290-8. Epub 2017/08/12. doi: 10.1111/jsr.12588. PubMed PMID: 28799194.

37. Tyrrell J, Mulugeta A, Wood AR, Zhou A, Beaumont RN, Tuke MA, et al. Using genetics to understand the causal influence of higher BMI on depression. Int J Epidemiol. 2019;48(3):834-48. Epub 2018/11/14. doi: 10.1093/ije/dyy223. PubMed PMID: 30423117; PubMed Central PMCID: PMCPMC6659462.

38. Cooper R, Popham M, Santanasto AJ, Hardy R, Glynn NW, Kuh D. Are BMI and inflammatory markers independently associated with physical fatigability in old age? Int J Obes (Lond). 2019;43(4):832-41. Epub 2018/05/26. doi: 10.1038/s41366-018-0087-0. PubMed PMID: 29795469; PubMed Central PMCID: PMCPMC6477893.

39. Okifuji A, Hare BD. The association between chronic pain and obesity. J Pain Res. 2015;8:399-408. Epub 2015/07/24. doi: 10.2147/jpr.S55598. PubMed PMID: 26203274; PubMed Central PMCID: PMCPMC4508090.

40. Toivanen AT, Heliövaara M, Impivaara O, Arokoski JPA, Knekt P, Lauren H, et al. Obesity, physically demanding work and traumatic knee injury are major risk factors for knee osteoarthritis—a population-based study with a follow-up of 22 years. Rheumatology. 2010;49(2):308-14. doi: 10.1093/rheumatology/kep388.

41. Wang W, Sun M, Xu Z, Qiu Y, Weng W. The low back pain in patients with hip osteoarthritis: current knowledge on the diagnosis, mechanism and treatment outcome. Annals of Joint. 2016;1(4).

42. Neogi T. The epidemiology and impact of pain in osteoarthritis. Osteoarthr Cartil. 2013;21(9):1145-53. Epub 2013/08/27. doi: 10.1016/j.joca.2013.03.018. PubMed PMID: 23973124; PubMed Central PMCID: PMCPMC3753584.

43. Burgess S, Thompson SG. Multivariable Mendelian randomization: the use of pleiotropic genetic variants to estimate causal effects. Am J Epidemiol. 2015;181(4):251-60. Epub 2015/01/30. doi: 10.1093/aje/kwu283. PubMed PMID: 25632051; PubMed Central PMCID: PMCPMC4325677.

44. McIlvane JM, Baker TA, Mingo CA. Racial Differences in Arthritis-Related Stress, Chronic Life Stress, and Depressive Symptoms Among Women With Arthritis: A Contextual Perspective. J Gerontol B Psychol Sci Soc Sci. 2008;63(5):S320-S7. doi: 10.1093/geronb/63.5.S320.

45. Ahn H, Weaver M, Lyon D, Choi E, Fillingim RB. Depression and Pain in Asian and White Americans With Knee Osteoarthritis. J Pain. 2017;18(10):1229-36. Epub 2017/06/18. doi: 10.1016/j.jpain.2017.05.007. PubMed PMID: 28619697; PubMed Central PMCID: PMCPMC5661986.

46. Sturgeon JA. Psychological therapies for the management of chronic pain. Psychol Res Behav Manag. 2014;7:115-24. Epub 2014/04/22. doi: 10.2147/prbm.S44762. PubMed PMID: 24748826; PubMed Central PMCID: PMCPMC3986332.

Table 1: Summary of data sets contributing to the Two-Sample MR analysis

| Phenotype | N | Ethnicity | Country/’s^b^ | Recruited | GWAS type | % UKBB^a^ | Phenotype definition | Phenotype description | Unit | Access site (code) | Ref |
| --- | --- | --- | --- | --- | --- | --- | --- | --- | --- | --- | --- |
| Knee OA | 396,054 | European (1% East Asian) | UK, IS, EE, JP, NL, GR, US, NO | 1984-2021 | Meta-analysis (fixed effect IVW) | 25 | Self-report, hospital diagnosed, radiographic | 16% cases | logOR | Musculoskeletal Knowledge Portal (KP.Format.GO.FILTER.GW.KneeOA.FULL.09052019.txt.gz) | [12] |
| Hip OA | 353,388 | European | UK, IS, EE, NL, GR, US, NO | 1984-2021 | Meta-analysis (fixed effect IVW) | 16 | Self-report, hospital diagnosed, radiographic | 10% cases | logOR | Musculoskeletal Knowledge Portal (KP.Format.GO.FILTER.GW.HipOA.FULL.09052019.txt.gz) | [12] |
| Hand OA | 303,789 | European | UK, IS, EE, NL, US, NO | 1984-2021 | Meta-analysis (fixed effect IVW) | 2 | Self-report, hospital diagnosed, radiographic | 7% cases | logOR | Musculoskeletal Knowledge Portal (KP.Format.GO.FILTER.GW.HandOA.FULL.09052019.txt.gz) | [12] |
| Knee OA (no UKBB^a^) | 298,274 | European (1% East Asian) | UK, IS, EE, JP, NL, GR, US, NO | 1984-2021 | Meta-analysis (fixed effect IVW) | 0 | Self-report, hospital diagnosed, radiographic | 14% cases | logOR | NA | [12] |
| Hip OA (no UKBB^a^) | 296,748 | European | UK, IS, EE, NL, GR, US, NO | 1984-2021 | Meta-analysis (fixed effect IVW) | 0 | Self-report, hospital diagnosed, radiographic | 8% cases | logOR | NA | [12] |
| Hand OA (no UKBB^a^) | 296,202 | European | UK, IS, EE, NL, US, NO | 1984-2021 | Meta-analysis (fixed effect IVW) | 0 | Self-report, hospital diagnosed, radiographic | 7% cases | logOR | NA | [12] |
| Depression | 807,553 | European | UK, US | 2006-2016 | Meta-analysis (fixed effect IVW) | 45 | Self-report | 31% cases | logOR | IEU Open GWAS project (ieu-b-102) | [15] |
| Tiredness | 449,019 | European | UK | 2006-2010 | Primary GWAS | 100 | Self-report | Tired: Not at all (46%), Several days (39%), More than half the days (6%), Every day (6%) | Per unit change | IEU Open GWAS project (ukb-b-929) | [16] |
| Multisite Chronic Pain | 387,649 | European | UK | 2006-2010 | Primary GWAS | 100 | Self-report | Sites of pain: 0 (56%), 1 (24%), 2 (12%), 3 (5%), 4 (2%), 5 (0.7%), 6 (0.2%), 7 (0.03%) | Per unit change | University of Glasgow Enlighten (chronic_pain-bgen.stats.gz) | [17] |
| Irritable Bowel Syndrome | 486,601 | European | UK, US, IT, NL, EE, SE, BE, NO, DE | 1995-2016 | Meta-analysis (fixed effect IVW) | 69 | Self-reported, hospital diagnosed | 11% cases | logOR | NHGRI-EBI GWAS catalog (GCST90016564) | [18] |
| Gout | 456,390 | European | UK | 2006-2010 | Primary GWAS | 100 | Hospital diagnosed | 1% cases | logOR | IEU Open GWAS project (ukb-b-13251) | [16] |

1. UKBB, UK Biobank
2. Standard two letter country codes were used to describe each country included in the study

Figure 1: MR analysis for causality from OA to comorbidities

GO, Genetics of Osteoarthritis, GWAS, Genome Wide Association Study, OA, Osteoarthritis, MRC-IEU, Medical Research Council-Integrative Epidemiology Unit, IVW, Inverse-Variance Weighted (analysis)

Figure 2: MR analysis for causality from comorbidities to OA

GO, Genetics of Osteoarthritis, GWAS, Genome Wide Association Study, OA, Osteoarthritis, MRC-IEU, Medical Research Council-Integrative Epidemiology Unit, IVW, Inverse-Variance Weighted (analysis)

Figure 3: Causal estimates between comorbidities and Knee OA

1. OR, Odds Ratio, PUC, Per Unit Change, CI, confidence interval, MCP, multisite chronic pain, IBS, irritable bowel syndrome, OA, osteoarthritis
2. Darker line and text mean p < 0.05 for both main and sensitivity

Figure 4: Causal estimates between comorbidities and Hip OA

1. OR, Odds Ratio, PUC, Per Unit Change, CI, confidence interval, MCP, multisite chronic pain, IBS, irritable bowel syndrome, OA, osteoarthritis
2. Darker line and text mean p < 0.05 for both main and sensitivity

Figure 5: Causal estimates between comorbidities and Hand OA

1. OR, Odds Ratio, PUC, Per Unit Change, CI, confidence interval, MCP, multisite chronic pain, IBS, irritable bowel syndrome, OA, osteoarthritis
2. Darker line and text mean p < 0.05 for both main and sensitivity

Figure 6: Average Causal Effects between OA and comorbidities

1. OR, Odds Ratio, PUC, Per Unit Change, CI, confidence interval, MCP, multisite chronic pain, IBS, irritable bowel syndrome, OA, osteoarthritis
2. Darker line and text mean p < 0.05 for both main and sensitivity

Figure 7: Multisite chronic pain SNP effects on OA

1. OA, Osteoarthritis, GO, Genetics of Osteoarthritis, MCP, Multisite Chronic Pain, IVW, Inverse Variance Weighted
2. The scale of the forest plots is untransformed logOR
